# St. George's Hospital in Danger

**Published:** 1904-07-09

**Authors:** 


### The Hospital.

A Journal of

The Medical Sciences and Hospital Administration.

Vol. XXXVI.—No. 928. JULY 9, 1904.

### St. George's Hospital in Danger.

We have refrained from dealing with the question of the site of St. George's Hospital, feeling that it was one which the governors must decide for themselves. As matters stand at present St. George's Hospital is committed to an expenditure of £300,000 at least upon the present site, and where this money is to come from it is impossible to say. The seriousness of the position, in view of the report which was presented to the governors by the honorary medical staff, must be apparent to everybody who reads between the lines. The hospital and school buildings as they exist to-day are inadequate for the purpose; many of them are out of date, and some of them, as indicated in a letter we recently published, cannot much longer be used for hospital purposes. The rebuilding of the entire hospital, as the staff have wisely demanded, upon an approved plan embracing the whole site must entail the closing of the hospital for a period of about two years. There is, therefore, a twofold difficulty to be faced: the indefinite delay in rebuilding due to the absence of funds and the facts that the hospital at present costs more per bed than it should cost, owing to the absence of perfectly planned buildings, and the dangers to the patients and the school from the imperfections already referred to.

It has been a matter of public comment in the press that the report of the committee to the governors was indefinite; that although the committee sat for more than a year it did not produce any practical results; and that the position in which matters are left at present is neither good for the hospital nor satisfactory to the public on whose goodwill the institution depends mainly for its continuance. The explanation is not far to seek. We understand that the original committee consisted of a majority of the older governors, who, mainly on sentimental grounds, were opposed to the sanction of any scheme for the removal of the hospital to a new site. It was only natural therefore that every step should be taken to delay the proceedings and to raise objection after objection to any proposals which aimed at bringing matters to a head by advertising the site and taking the necessary steps as a matter of business to ascertain its value and the amount of money which could actually be obtained for it in the most favourable circumstances. We will not blame those who are responsible for the practical failure of the committee in the task which was delegated to them by the governors of St. George's Hospital, because sentiment has much to do with hospital support. Still we are bound to say that we consider they have taken upon themselves a grave responsibility, and that it rests with these governors to at once show the faith that is in them by subscribing, with the aid of their friends, the £300,000 to which they have committed the governors in order to build a modern hospital for 300 beds upon the existing site. If they fail to raise this money their names may go down to history as responsible for all the dangers which may arise.

St. George's is practically the last of the larger hospitals which retains the system of government by an open board. That system has proved such a failure in practice that it has now almost entirely disappeared. Every hospital which desires to secure vigorous life and the best of administration has wisely abolished this system, and the dangers attendant upon it are well illustrated by the present position of St. George's Hospital, as shown by the agenda of business to be submitted to the quarterly general court of the governors on July 8th next. The theory of the open board is that every governor has the right to attend and take part in the administration of the hospital's affairs. In practice experience shows that this system means that the management falls into a very few hands, and that the general interest of the governors lessens until it practically ceases to exist. At the present time nine governors constitute a quorum at any quarterly or special court, and that quorum is raised to 11 governors when the annual accounts are presented. It is now proposed to reduce this inadequate quorum from nine to seven, and to abolish the second quorum altogether. Thus the affairs of St. George's Hospital for the future are to be placed at the mercy of any seven governors who happen to be present at a quarterly court. Such a state of affairs shows that steps should be taken without delay to amend the laws and constitution of St. George's Hospital and to bring them into line with the best modern traditions of hospital government. Unless this is done we venture to predict that the interest of the governors in the welfare of this hospital, which is at present unfortunately inadequate, will grow less and less until the very existence of this great charity may be threatened. Our sympathies are entirely with the medical staff of St. George's Hospital who showed their good judgment by insisting at the special court that no further money should be expended upon buildings on any portion of the existing site except upon perfected and complete plans, first finally agreed to, which will provide for the erection of an entirely new hospital.

